# Supplementary material for: Iterative improvement in the automatic modular design of robot swarms
Source: PeerJ Comput Sci. 2020 Dec 7;6:e322. doi: 10.7717/peerj-cs.322 (PMC7924708; doi:10.7717/peerj-cs.322)
Supplement: Supplemental Information 3 [file peerj-cs-06-322-s003.zip › argos3/doc/api/standalone/a00509_source.html]

ARGoS: plugins/robots/generic/control\_interface/ci\_proximity\_sensor.h Source File


- Main Page
- Related Pages
- Namespaces
- Classes
- Files

- File List
- File Members

# plugins/robots/generic/control\_interface/ci\_proximity\_sensor.h

Go to the documentation of this file.

```
00001 
00007 #ifndef CCI_PROXIMITY_SENSOR_H
00008 #define CCI_PROXIMITY_SENSOR_H
00009 
00010 namespace argos {
00011    class CCI_ProximitySensor;
00012 }
00013 
00014 #include <argos3/core/control_interface/ci_sensor.h>
00015 
00016 namespace argos {
00017 
00018    class CCI_ProximitySensor : public CCI_Sensor {
00019 
00020    public:
00021 
00022       virtual ~CCI_ProximitySensor() {}
00023 
00024       const std::vector<Real>& GetReadings() const;
00025 
00026 #ifdef ARGOS_WITH_LUA
00027       virtual void CreateLuaState(lua_State* pt_lua_state);
00028 
00029       virtual void ReadingsToLuaState(lua_State* pt_lua_state);
00030 #endif
00031 
00032    protected:
00033 
00034       std::vector<Real> m_tReadings;
00035 
00036    };
00037 
00038 }
00039 
00040 #endif
```

---

Generated on 10 Jul 2018 for ARGoS by 
 1.6.1
